# Matriptase processing of APLP1 ectodomain alters its homodimerization

**DOI:** 10.1038/s41598-020-67005-6

**Published:** 2020-06-22

**Authors:** Erwan Lanchec, Antoine Désilets, François Béliveau, Cloé Fontaine-Carbonneau, Andréanne Laniel, Richard Leduc, Christine Lavoie

**Affiliations:** Department of Pharmacology-Physiology, Faculty of Medicine and Health Sciences, Université de Sherbrooke, Sherbrooke, QC J1H5N4 Canada

**Keywords:** Proteases, Alzheimer's disease

## Abstract

The amyloid beta peptide (Aβ) is derived from the amyloid precursor protein (APP) by secretase processing. APP is also cleaved by numerous other proteases, such as the type II transmembrane serine protease matriptase, with consequences on the production of Aβ. Because the APP homolog protein amyloid-like protein 1 (APLP1) shares similarities with APP, we sought to determine if matriptase also plays a role in its processing. Here, we demonstrate that matriptase directly interacts with APLP1 and that APLP1 is cleaved *in cellulo* by matriptase in its E1 ectodomains at arginine 124. Replacing Arg124 with Ala abolished APLP1 processing by matriptase. Using a bioluminescence resonance energy transfer (BRET) assay we found that matriptase reduces APLP1 homodimeric interactions. This study identifies matriptase as the first protease cleaving APLP1 in its dimerization domain, potentially altering the multiple functions associated with dimer formation.

## Introduction

Alzheimer’s disease (AD) is a neurodegenerative disease characterized by a progressive and accelerated loss of neurons, leading to cognitive disorders and is currently the most common dementia^1^. Accumulation of extracellular amyloid beta (Aβ) whether in the form of plaques, oligomers or soluble monomers is a fundamental hallmark of AD^2,3^. In the pathogenic amyloidogenic pathway, successive APP cleavages by β- and γ-secretase results in the production of Aβ^4^. Recent treatment strategies targeting elements of the amyloidogenic pathway have failed to slow the progression of symptoms. Therefore, a better understanding of the mechanisms involved in AD is needed and several teams have focused on the physiological role and biosynthesis/processing of APP family members.

Amyloid-like protein 1 (APLP1) is part of the same family and is homologous to APP^5^. According to the Human Protein Atlas^6,7^, APLP1 is enriched in the human brain while APP is ubiquitously expressed, consistent with data obtained in mice^8^. APP and APLP1 are type I transmembrane proteins sharing conserved luminal E1 and E2 domains^5^. The E1 domain, rich in cysteines, is comprised of two subdomains, a growth factor-like subdomain (GFLD) that binds heparin and that stimulates neurite growth, as well as a CuBD subdomain that binds Cu and Zn ions^9^. The E2 domain forms an antiparallel dimer and binds heparin in its dimeric form^10^. Finally, the C-terminal domains of these proteins contain a YENPTY motif that serves as an endocytosis signal^11^. Although both proteins can be cleaved by secretases, the Aβ sequence is only found in APP^12,13^. APP and APLP1 are both involved in neuronal differentiation, synaptogenesis, neurite growth, and synaptic plasticity^14–16^.

APP and APLP1 are known to form homo- and heterodimers^17^, which are in part dependent on the conserved E1 domain^18^. These dimeric interactions occur at the plasma membrane on a single cell (*cis* interaction) but also occur between transmembrane proteins of adjacent cells (*trans* interaction) ^19–21^. APP/APLP1 interactions promote cell adhesion in a homo- and heteromeric fashion^17^. This process is triggered by heparin binding to the E1 domain followed by induction of E2 domain dimerization. Furthermore, when the ability of APP to form dimers is impaired, it influences the ability of BACE1 secretase to cleave APP, resulting in decreased Aβ peptide production^22–25^.

Proteases present at the plasma membrane as well as in the extracellular space play important roles in development, homeostasis and tissue remodeling^26^. The plasma membrane/extracellular enzyme matriptase is a type II transmembrane serine protease (TTSP) encoded by the suppression of tumorigenicity-14 gene (ST14)^27^. This protease undergoes autoactivation at the plasma membrane where it can cleave various substrates^12,28–31^ or be released into the extracellular medium as a shed and enzymatically active form^32^. Although the expression of matriptase was documented to occur predominantly in epithelial cells of different organs, a growing number of studies have reported expression of matriptase in the brain and/or suggest a role for matriptase in the central nervous system (CNS)^33–37^. Indeed, unregulated matriptase activity disrupts neural tube closure in embryonic mice^33^ while its expression in neuronal progenitor cells promotes cell migration and neuronal differentiation^34^. Other studies observed an increase of matriptase transcript levels in a mouse model of AD, especially in activated microglia around amyloid plaques^36,37^.

We have recently shown that matriptase mRNA is expressed in different regions of human brain, with an enrichment in neurons and that it is also present at the protein level in differentiated neurons derived from human induced pluripotent stem cells (hiPSCs)^38^. Moreover we showed that matriptase cleaves the three APP isoforms in the E1 domain at residue 102^38^. This cleavage, although distant from the Aβ sequence, alters the production of Aβ peptide in cellular assays. Since the E1 domain is conserved among members of the APP family and is important for dimerization, we investigated the possibility that matriptase cleaves APLP1 and alters the dimerization/heterodimerization process.

In this study, we show that matriptase interacts with and cleaves APLP1 at a specific residue in its E1 ectodomain. Using a BRET-based assay, we show that addition of matriptase to cells expressing APLP1 disrupts the protein’s ability to form homodimers. These events may have important consequences on the physiological and pathological functions of APP family members.

## Results

### **Matriptase interacts with APLP1**

**Figure 1:**
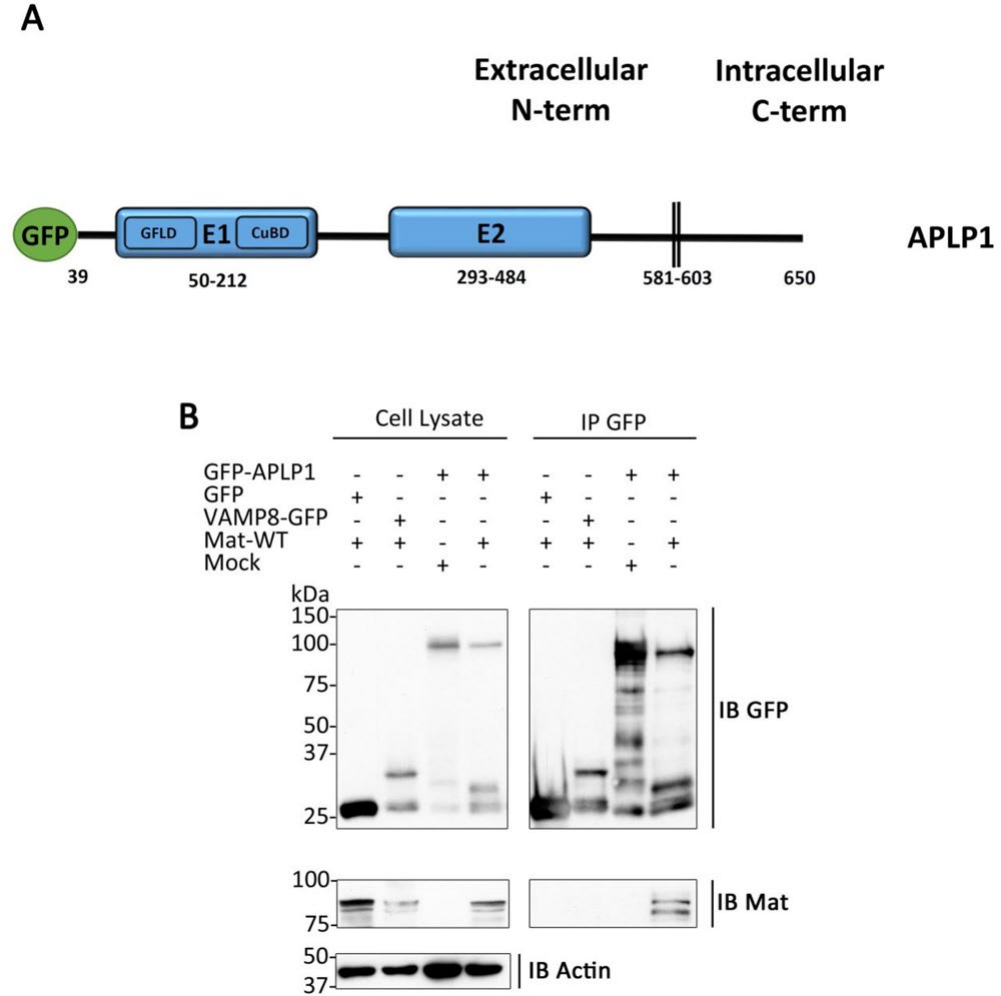
Matriptase interacts with APLP1. (**A**) Schematic representation of the GFP-tagged isoforms of APLP1. The elements constituting APLP1 are shown, including the E1 and E2 domains, growth factor-like (GFLD) and copper binding (CuBD) subdomains and transmembrane domain (**B**) Lysate of HEK293T cells transfected with matriptase (Mat-WT), GFP-tagged APLP1 (GFP-APLP1), luminal GFP (GFP), VAMP8-GFP or an empty vector (Mock) were immunoprecipitated (IP) with GFP-Trap beads and then immunoblotted (IB) with anti-matriptase, anti-actin or anti-GFP antibodies to detect matriptase, actin, GFP, VAMP8 and APLP1, respectively (n = 3). Cropped blots are displayed. Full length blots are displayed in Supplementary Fig. S1.

In order to explore the possibility that matriptase interacts with APLP1, an APLP1 construct tagged at its N-terminus (extracellular/luminal side) with GFP (containing the signal peptide of containing the mannose-6-phosphate receptor signal peptide) was expressed in HEK293T cells (Fig. 1A) together with matriptase. Immunoblot analysis of cell lysates reveals that GFP-APLP1 is detected as a major 120 kDa form (Fig. 1B), whereas matriptase is detected as a doublet at 95 kDa, which reflects the presence of the full-length 855 amino acid protein and a constitutively processed form at glycine 149^38–40^. Furthermore, matriptase co-immunoprecipitates with GFP-APLP1 but does not co-immunoprecipitate with GFP expressed in the lumen of compartments (GFP containing a signal peptide). The specificity of this interaction was further confirmed using VAMP8-GFP, a transmembrane protein tagged at its luminal side with GFP (Fig. 1). Finally, a specific co-immunoprecipitation between matriptase and APLP1 was also observed using an APLP1 construct tagged at its C-terminus with Flag (Supplementary Fig. S2). Together, these results indicate that matriptase interacts *in cellulo* with APLP1.

**Figure 2:**
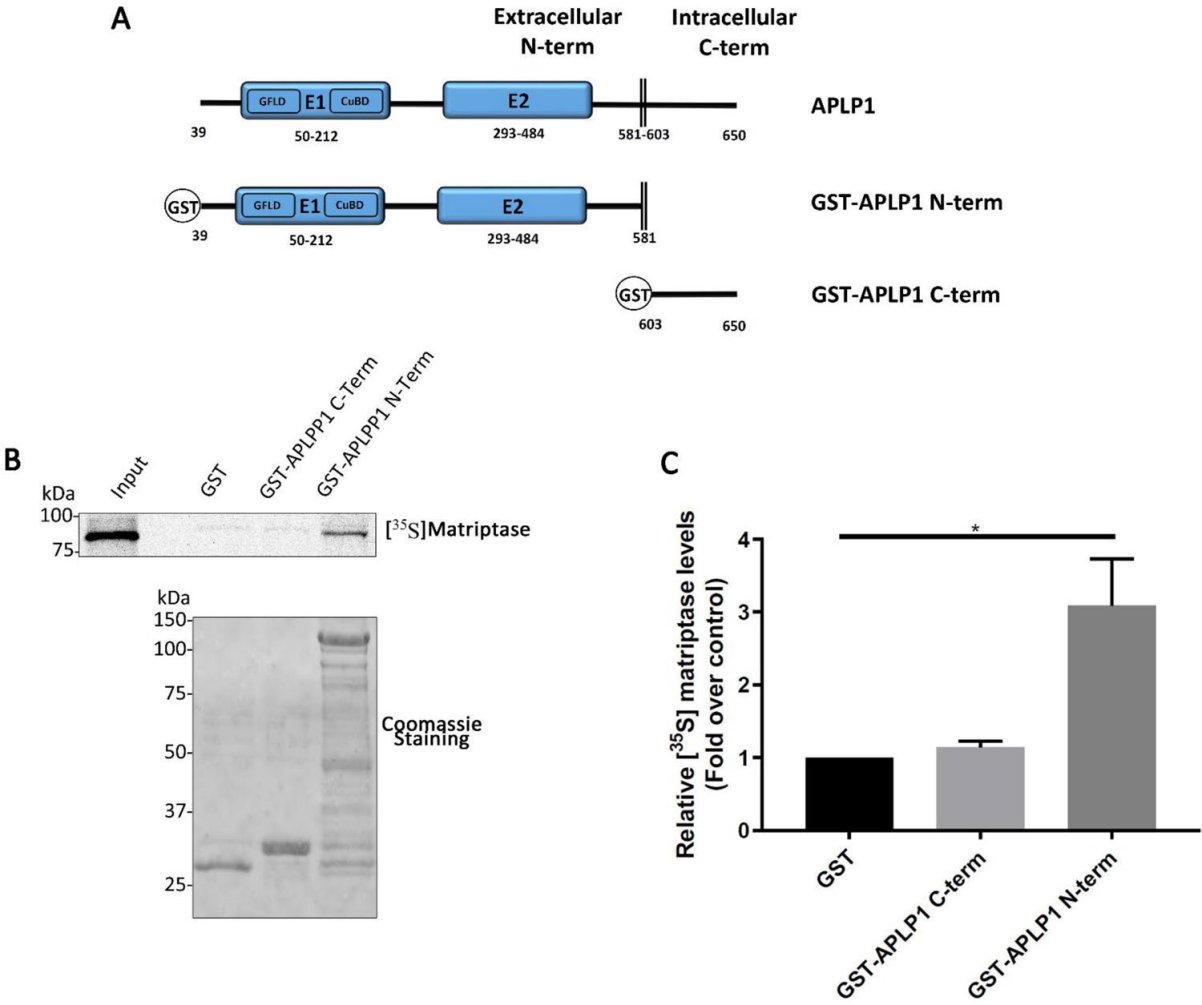
*In vitro* interaction of matriptase with the ectodomain of APLP1. (**A**) Schematic representation of the GST-tagged APLP1 deletion mutants used to determine the matriptase binding domain. (**B**) GST-tagged APLP1 mutants described in (A) and GST protein (10 μg) were immobilized on glutathione beads and incubated with *in vitro* translated ^35^S-labeled matriptase. Bound proteins were separated by SDS-PAGE and detected by autoradiography. GST proteins were detected with Coomassie blue staining. Input = 2.5% of the total *in vitro* translated product (n = 3). (C) A one sample t- test with a hypothetical value set to 1 on the densitometric analysis of Fig. 2B was applied. There is a statistical difference between GST alone and GST-APLP1 N-term but not between GST alone and GST-APLP1 C-term (p < 0.05). Cropped autoradiography films are displayed. Full length autoradiography films are displayed in Supplementary Fig. S3.

To determine whether the co-immunoprecipitation observed between APLP1 and matriptase is a consequence of direct interactions, we then performed *in vitro* GST pull-down experiments (Fig. 2). N-terminal GST chimeric constructs corresponding to the extracellular or intracellular domains of APLP1 (Fig. 2A) were expressed in bacteria. The purified recombinant GST proteins were then incubated with *in vitro* translated,^35^S-labeled matriptase and GST-bound proteins were detected by autoradiography. A robust signal was obtained when matriptase was incubated with the GST-APLP1 N-term protein while a weak signal was detected with GST alone or with GST-APLP1 C-term (Fig. 2B). Densitometric analysis revealed a statistically significative difference using GST-APLP1 N-term protein when compared to GST alone (Fig. 2C). These results demonstrate that a direct interaction occurs between matriptase and the ectodomain of APLP1 but not with GST-APLP1 C-term.

### ***In cellulo*cleavage of APLP1 by matriptase**

**Figure 3:**
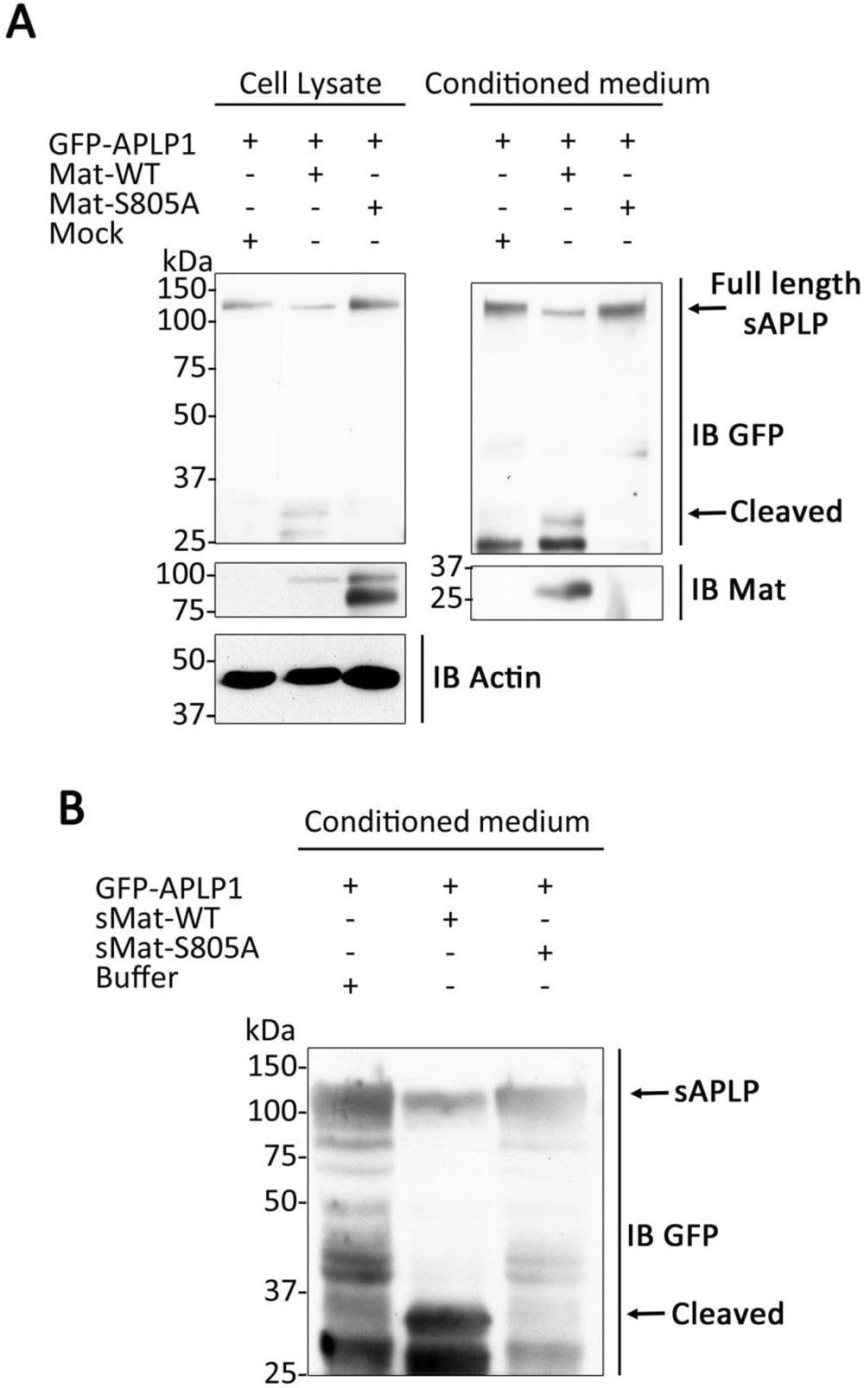
Matriptase cleaves APLP1 *in cellulo*. (**A**) HEK293T cells were transfected with wild-type matriptase (Mat-WT), a catalytically inactive matriptase mutant (Mat-S805A) or empty vector (mock) together with GFP-tagged APLP1. Lysates and conditioned media were immunoblotted (IB) with anti-matriptase, anti-actin or anti-GFP antibody to detect matriptase, actin, APLP1 and APLP1 fragments (n = 3). Note the GFP-tagged APLP1 fragment (cleaved) of 33 kDa in cell lysate and medium (arrow). (**B**) HEK293T cells transfected with GFP-tagged APLP1 were incubated without (buffer) or with 5 nM of recombinant soluble WT matriptase (sMat-WT) or catalytically inactive matriptase (sMat-S805A). Conditioned media were immunoblotted as described in (A) (n = 3). Full length blots are displayed in Supplementary Fig. S4.

When APLP1 was co-transfected with matriptase, a GFP-APLP1 fragment migrating at 33 kDa was detected in the cell lysate (Fig. 1B). This 33-KDa fragment would correspond to the GFP tag (25 kDa) and a portion of the N-terminal domain of APLP1 (8 kDa). To further characterize cleavage of APLP1 by matriptase, HEK293T cells were transfected with GFP-APLP1 in combination with matriptase or with a catalytically inactive form of matriptase (S805A). Physiologically, substrate cleavage by matriptase occurs at the plasma membrane or in the extracellular medium^32,41^. Therefore, cell lysates and culture medium were harvested to detect APLP1 cleavage products. The GFP-tagged APLP1 fragment of 33 kDa was detected in both cell lysates conditioned medium of cells transfected with wild-type matriptase but not in cells transfected with the inactive matriptase S805A (Fig. 3A). This result demonstrates that APLP1 is processed by catalytically active matriptase into soluble forms found in the media of transfected cells. This cleavage by catalytically active matriptase was also validated by co-expressing HAI-1, the physiological inhibitor of matriptase. Indeed, no cleavage products are detected when GFP-APLP1 and matriptase are co-transfected with HAI-1 (Supplementary Fig. S5). Finally, when matriptase-2, a protease closely related to matriptase, is co-expressed with GFP-APLP1, no cleavage products are observed (Supplementary Fig. S6) supporting the idea of a specific cleavage by matriptase.

Because matriptase is shed in the extracellular space as a soluble form^32,41^, we investigated whether exogenous addition of an enzymatically active soluble form of matriptase cleaves GFP-APLP1 expressed in HEK293T cells. A GFP-tagged APLP1 fragment of 33 kDa was detected in the conditioned culture medium of GFP-APLP1 expressing cells incubated with recombinant soluble active matriptase but not with soluble inactive matriptase S805A (Fig. 3B). Altogether, these results suggest that active matriptase can cleave GFP-APLP1 at the cell surface to generate soluble APLP1 forms.

### **Identifying matriptase cleavage sites on APLP1**

**Figure 4:**
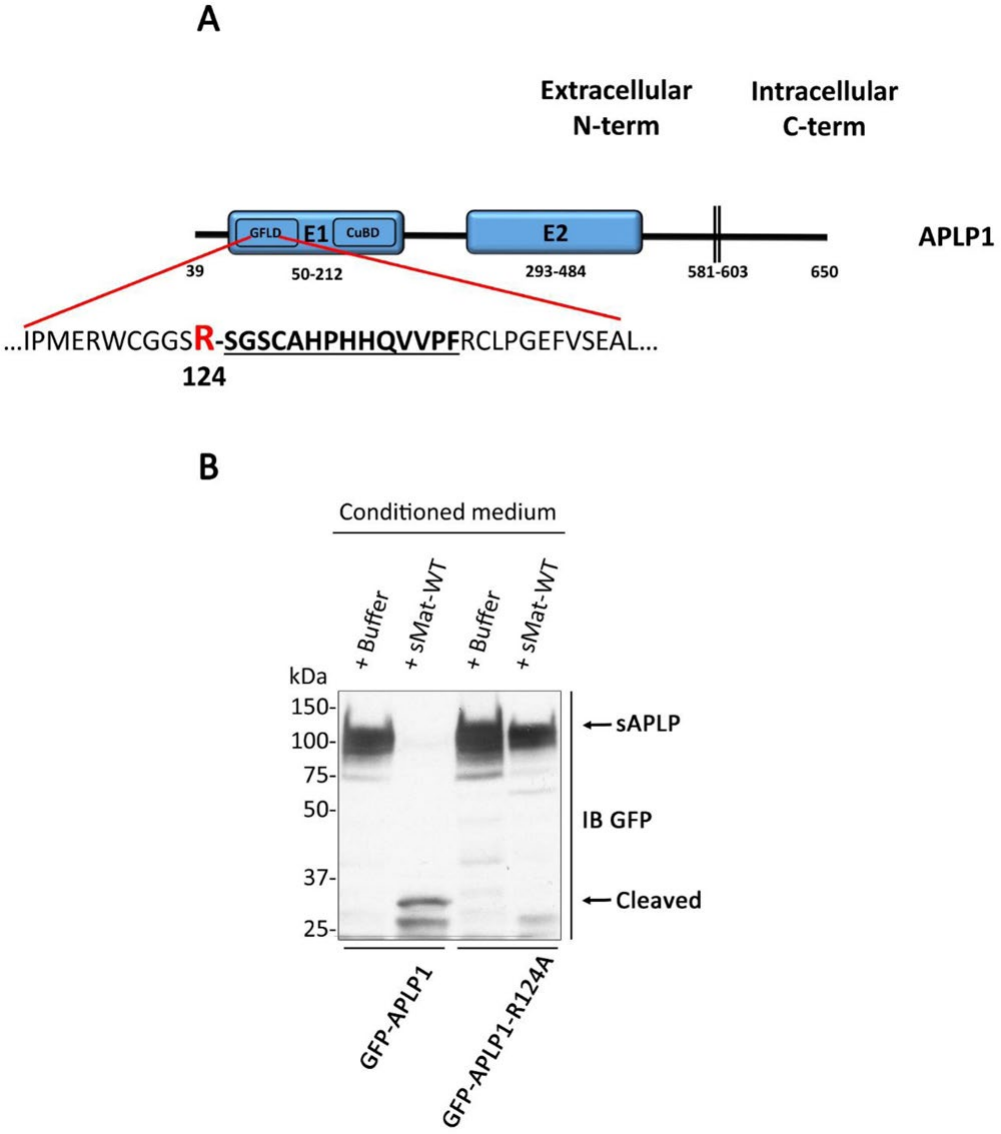
Matriptase cleaves APLP1 at arginine 124. (**A**) Schematic representation of the position of the Arg cleaved by matriptase in the ectodomain of APLP1 tagged with GST that was used to determine matriptase cleavage site (see Supplementary Fig. S7). (**B**) HEK293T cells transfected with GFP-tagged APLP1 wild-type (GFP-APLP1) or in which Arg-124 was mutated to Ala (GFP-APLP1-R124A) were incubated without (buffer) or with 5 nM of recombinant WT matriptase (sMat-WT). Conditioned media were immunoblotted (IB) with anti-GFP antibody to detect soluble APLP1 (sAPLP) and APLP1 fragments (n = 3). Note the absence of the 33 kDa GFP-tagged APLP1 fragment (cleaved) in the GFP-APLP1-R124A lanes.

To identify the precise matriptase cleavage sites within APLP1 extracellular domain, mass spectrometry (MS) analysis was performed on APLP1 fragments generated following *in vitro* incubation of purified GST-APLP1 ectodomains with or without recombinant soluble matriptase. A major cleavage site was found at arginine 124 of APLP1 (Fig. 4A and Supplementary Fig. S7). This cleavage site is predicted to yield N-terminal fragments with molecular weights of 9.6 kDa. Taking into account the molecular weight of the N-terminal GFP tag (25 kDa), this is consistent with the molecular weight of the fragment observed in Fig. 3 (33 kDa). Interestingly, similar to APP^38^, the matriptase cleavage site was found in the E1 domain of APLP1. In order to confirm this cleavage site, Arg-124 was mutated to Ala (R124A) in GFP-tagged APLP1. HEK293T cells expressing either wild-type GFP-APLP1 or GFP-APLP1-R124A were then incubated with (or without) soluble matriptase and analyzed for the presence of APLP cleavage products in the conditioned medium (Fig. 4B). Expression of the R124A mutant completely abolished the formation of the GFP-APLP1 cleaved fragment indicating that this mutant is resistant to cleavage by matriptase. These results confirmed Arg-124 as the primary matriptase cleavage site in APLP1. Taken together, these results indicate that, similarly to APP, matriptase cleaves in the E1 domain of APLP1.

### **Matriptase processing of APLP1 alters its homodimerization**

**Figure 5:**
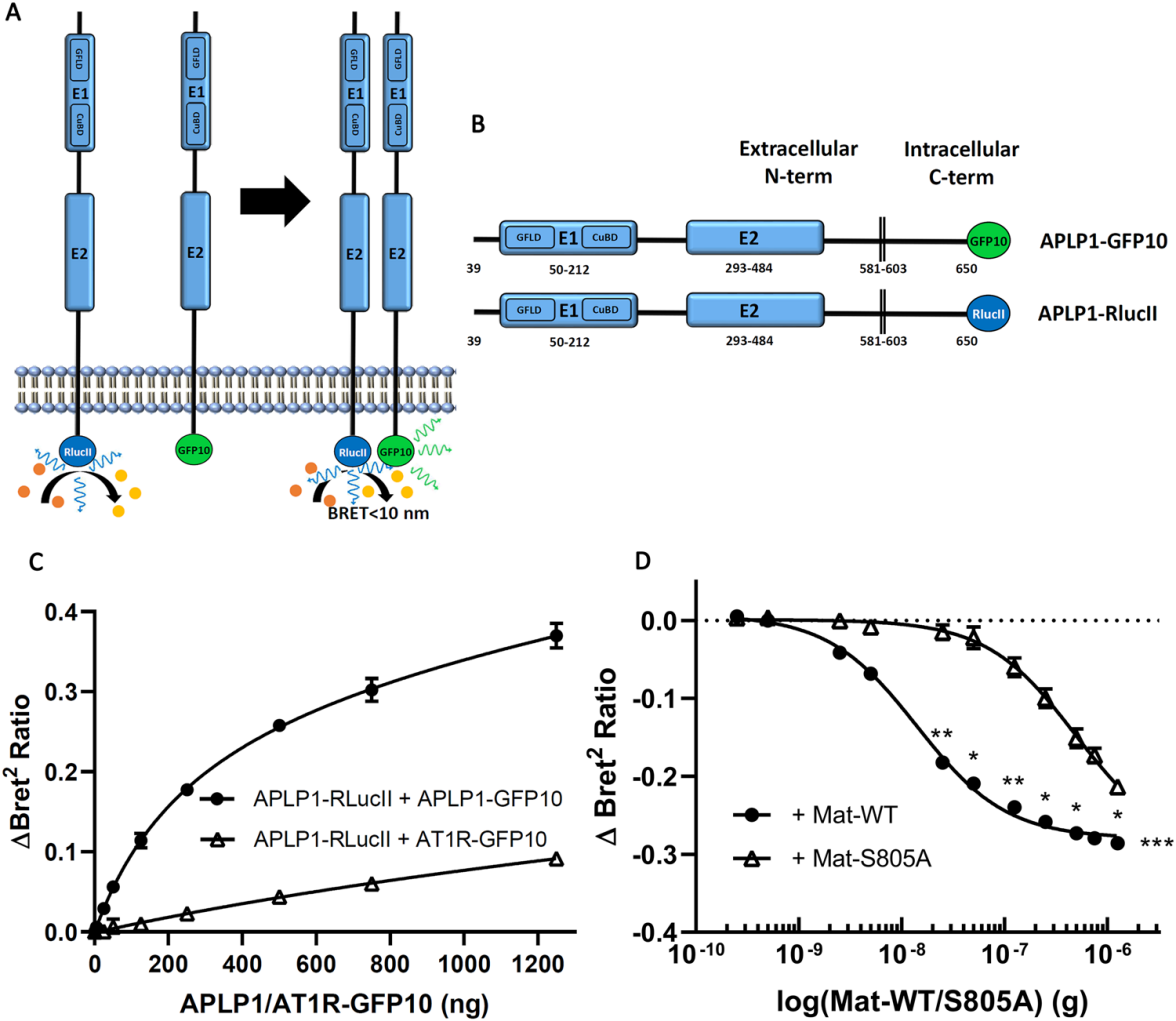
Matriptase disturbs APLP1 homodimeric interaction. (**A**) Schematic representation of the BRET homodimerization assay. When GFP10 (acceptor molecule) is in close vicinity (<10 nm) to RlucII (donor molecule) in the presence of its substrate coelenterazine, a BRET signal is generated. (**B**) Schematic representation of the constructs APLP1-GFP10 and APLP1-RLucII used to study homodimeric interactions of APLP1. (**C**) HEK293T cells transfected with RLucII-tagged APLP1 (100 ng) and an increasing DNA quantity of GFP10-tagged APLP1 or GFP10-tagged AT1R were incubated with coelenterazine and signals were measured with a plate reader (n = 3 for each curve). (**D**) HEK293T cells transfected with GFP10-tagged APLP1 and RLucII-tagged APLP1 were co-transfected either with an increasing DNA quantity of matriptase (Mat-WT) or matriptase S805A (Mat-S805A). Cells were incubated with coelenterazine and signals were measured with a plate reader (n = 3 for each curve). A two-way ANOVA with a Sidak’s multiple comparisons test was applied on the data set of each curve. There is a statistical difference between Mat-WT and Mat-S805A (p = 0.0003).

APP family members can form homotypic and heterotypic *cis* and *trans* dimers mainly through their N-terminal E1 domains^17^. Given that we defined the matriptase cleavage site in the E1 domain of APLP1, we next examined whether this cleavage impairs its dimerization. We set up a novel Bioluminescence Resonance Energy Transfer (BRET2) assay to quantify APLP1 *cis*-homodimerization^42^. In this assay, when GFP10 (acceptor molecule) is in close vicinity to Renilla luciferase II (RlucII, donor molecule) in the presence of its substrate coelenterazine, a BRET2 signal is generated and can be measured (Fig. 5A). To detect APLP homodimerization, GFP10 and RLucII moieties were fused to the C terminus of APLP1 (Fig. 5B). The BRET2 pair, APLP1-GFP10/-RLucII exhibits a saturation curve for a fixed concentration of donor (RLucII) and an increasing concentration of the acceptor, GFP10 (Fig. 5C). This result reflects a typical saturation profile consistent with a specific interaction between APLP1 monomers. On the other hand, this saturation plateau is not reached using an increasing concentration of the plasma membrane expressed angiotensin II type 1 receptor, (AT1R-GFP10) that does not interact with APLP1. These results confirm the homodimeric interaction for APLP1 proteins and validate our BRET2 approach to monitor dimerization.

We then assessed the impact of matriptase cleavage on the homodimeric interaction of APLP1 by expressing fixed concentrations of the APLP1 BRET constructs with increasing concentration of transfected active (WT) or inactive (S805A) matriptase. BRET2 signal reduction is expected if dimerization is impaired. Indeed, a BRET2 signal decrease was detected with increasing concentrations of active (WT) or inactive (S805A) matriptase for APLP1 homodimers (Fig. 5D). This suggests that interaction between matriptase and APLP1 affects the ability of APLP1 to remain as dimers. The effect on dimerization seen with active and inactive matriptase is in accordance with co-immunoprecipitation assays demonstrating an interaction between APLP1 and both matriptase forms. Furthermore, analyzing the dose dependent BRET2 response curves, we observed a leftward shift of the curves generated with active matriptase compared to the inactive protease. These results suggest that the catalytically active matriptase was more potent at disrupting APLP1 homodimers than inactive matriptase, probably due to its ability to cleave the E1 domain of APLP1. Statistical analysis of the data set of each curve show a statistical difference between the two curves.

## Discussion

For several years, research efforts have focused on the general understanding of the pathophysiology of AD and on APP processing for the development of therapeutic approaches. In this context, several novel proteases have been recently identified to cleave APP and to alter Aβ production^43^. However, much less is known about the processing of APP homologs APLPs and whether their proteolytic fragments have biological functions in the CNS or in peripheral tissues. Here, we identify matriptase as a novel protease that cleaves APLP1 at a specific residue in its E1 ectodomain, which impacts its ability to form homodimers, a key molecular event regulating its functions.

Matriptase is one of the most studied TTSPs and its expression was considered limited to epithelial cells. However, several recent studies have shown it is expressed in human brain regions^38^, neuronal progenitor cells^34^, microglia, activated astrocytes around amyloid plaque and neuronal IPSC cells^36–38,44^. We and others previously showed that matriptase cleaves APP^38,45^, a novel substrate for matriptase. Here, we find that matriptase interacts with and cleaves APLP1. Physiologically, this interaction and cleavage could occur in the brain where expression of matriptase is low but expression of APLP1 is high as well as in peripheral tissues, such as pancreas, where both proteins are expressed^6^.

We observed that matriptase interacts with and cleaves APLP1 *in cellulo*. Interaction with matriptase has been confirmed *in vitro* for APLP1’s ectodomain. Accordingly, mass spectrometry analysis and mutagenesis studies have identified Arg124 within the sequence GSRR^124^↓SGSC in the E1 domain of the extracellular region of APLP1 as the major matriptase cleavage site. To our knowledge, this specific arginine has never been identified as a processing site in APLP1 by other proteases. Interestingly, we previously reported that matriptase cleaves at Arg102 of APP’s E1 domain^38^. Sequence alignment of matriptase cleavage sites of APP and APLP1 highlighted their location at the same position in the GFLD region of their E1 domains (Supplementary Fig. S8), indicating that this well-exposed region is favorable for matriptase processing. In summary, we now report that both APP and APLP1 are cleaved by matriptase in a conserved region of their E1 domains.

Compared to APP, much less is known about the proteolytic cleavage of APLPs and the functional consequence of its processing. APLPs, like APP, undergo shedding of their ectodomain by α- and β-secretases, followed by γ-secretase-mediated intramembrane proteolysis^46–48^. Although the physiological role of the shed forms of APP is starting to emerge, the function of shed APLPs is less well defined. However, different forms of soluble APLPs have been detected in human cerebrospinal fluid^49–51^ and it has been shown that a soluble N-terminal fragment of the closely related member APLP2 binds Death receptor 6 (DR6)^52^. In addition to secretases, another protease, rhomboid protease RHBDL4, cleaves multiple sites within APLP ectodomains resulting in several N- and C-terminal fragments^53^. These cleavages occur in the lumen of the endoplasmic reticulum and regulate cell surface APLP levels^53^. Matriptase is thus one of the few proteases reported to cleave APLP1 ectodomains, potentially generating fragments that can have biological activities or altering processing by other proteases.

We report here that inactive matriptase can alter APLP1 dimerization and furthermore, that the active form is even more potent at disrupting this dimer. This suggests that modulation of APLP1 dimerization involves two distinct mechanism i.e. through direct protein-protein interaction and proteolytic cleavage in the E1 domain. The E1 domain of APP family members has been previously reported to mediate homo- and heterophilic interaction of APP/APLPs^17,23^. Deleting the GFLDs of APP and APLP2 inhibited *cis* homo- and hetero-dimerization of APP and APLP2 but only marginally affected APLP1 dimerization^23^, suggesting that the E2 domain is crucial for initiating APLP1 interaction^21^. The BRET homodimerization assay indicated that cleavage by matriptase in the GFLD region reduces the capacity of APLP1 to form *cis* homodimers. Although mass spectrometry analysis indicates that the main cleavage by matriptase occurs in the GFLD domain, we cannot exclude cleavage at other sites or that indirect effects of matriptase activity are also involved in this inhibition of APLPs *cis* interaction. Moreover, given that the E1 domain is also a key interface for APP/APLPs *trans* dimerization^17^, we expect that matriptase cleavage would affect *trans* interaction of APP family members. *Cis* and *trans* homo- and hetero-dimerization of APP family members has been involved in multiple functions including cell signaling^54^, neurite outgrowth^55^, neuronal cell adhesion^21^, trans-cellular adhesion^17^, synapse formation and function^15,56,57^, as well as Aβ peptides production^22,23,25^. Thus, membrane-bound or soluble matriptase cleavage of APP family members could regulate their functions by altering their capacity to dimerize in the brain or in peripheral tissues.

In conclusion, this study identifies matriptase as a novel APLP1 cleaving protease altering its ability to form dimers, an important feature that is known to affect its functions. Importantly, evaluating the impact of those events in a physiological context would provide novel insights into how APLP1 function is regulated *in vivo* and to which extent this regulation could influence pathological states.

## Methods

### Antibodies and reagents

Anti-GFP rabbit polyclonal antibodies (pAbs) and anti-human matriptase pAbs were purchased from Clontech Molecular Probes (Eugene, OR, USA), and Bethyl Laboratories (Montgomery, TX) respectively and were used as described previously^38^.

### BRET-based biosensor assays

HEK293T cells were transfected with indicated constructs and plated in 96-well white plates (50,000 cells/well). After 48 h, cells were then washed with buffer (10 mM Hepes, 1 mM CaCl_2_, 0.5 mM MgCl_2_, 4.2 mM KCl, 146 mM NaCl, 5.5 mM glucose, pH 7.4) and incubated with 5 μM coelenterazine 400 A for 10 minutes. Using a TECAN M1000 fluorescence plate reader, signals were measured. BRET ratio was calculated as the GFP10 emission over RLucII luminescence emission.

### Cell culture

HEK293T cells were kindly provided by Dr. Alexandra Newton (University of California, San Diego, CA, USA) and were grown in Dulbecco’s modified Eagle’s high glucose medium (Invitrogen) with 10% fetal bovine serum (FBS) (Hyclone Laboratories, Logan, UT, USA), 2 mM L-glutamine, 50 IU/mL penicillin and 50 µg/mL streptomycin (Wisent, St-Bruno, QC, Canada) as previously described^38^. HEK293T cells were transfected with Lipofectamine 2000 transfection reagent (Invitrogen), according to the manufacturers’ instructions

### Co-immunoprecipitation and immunoblotting

HEK293T cells were plated in 60-mm culture dishes and transfected with indicated constructs and co-immunoprecipitation assays were performed as previously described^38^. Briefly, after 48 h, cells were washed twice with PBS and lysed in 50 mM Tris buffer (pH 7.4) containing 150 mM NaCl, 1% triton x-100, and protease inhibitors for 1 h at 4 °C and were then centrifuged 20 min at 15,000 g. Supernatants were incubated with GFP-TrapA (Chromotek, Germany) overnight at 4 °C, washed with 50 mM Tris buffer (pH 7.4) containing 150 mM NaCl, 1% triton x-100, and protease inhibitors three times. Bound immune complexes were boiled in Laemmli sample buffer and proteins separated by SDS-PAGE. Immunoblotting was performed as described previously^38^.

### DNA constructs

Mammalian expression vectors APLP1 were kindly provided by Dr. Gerhard Multhaup (McGill University, Montreal). APLP1 was subcloned in pCMV5 downstream of the signal peptide (SP) of mannose-6-phosphate receptor and GFP (as previously described^38,58^) to produce an APLP1 tagged at its N-terminus (luminal/extracellular side) with GFP. Using this plasmid construction as a backbone, a SP-GFP control (GFP expressed in luminal compartment) was produced by introduction of a stop codon between GFP and APLP1 and the mutant GFP-APLP1-R124A was produced by substitution of an Arg to Ala at position 124 in GFP-APLP1 sequence. Both constructs were obtained using the QuikChange Lightning site-directed mutagenesis kit (Agilent Technologies). APLP1 containing the N-terminus (residues 39–581) and C-terminus (residues 597–650) coding sequences were subcloned in pET41a (Novagen). For BRET assays, APLP1 coding sequence was inserted upstream of GFP10 and RLucII in a pIRES-Hygromycin backbone using the NEBuilder HiFi DNA Assembly cloning kit as recommend by the manufacturer’s instructions (New England Biolabs, MA, USA). Recombinant matriptase (residues 596–855) construction used for bacterial expression (pQE30 vector, Qiagen, Mississauga, ON, Canada) and S805A-matriptase-pcDNA3.1 have been described previously ^39,59^. GFP-VAMP8 cDNA was kindly provided by Dr. Steve Jean (Université de Sherbrooke) and has been previously described^60^. All constructs were submitted to nucleotide sequencing before being used.

### **Glutathione S-transferase pull-down assays**

GST pull down assays were performed as described previously^38^. GST fusion proteins expressed in *Escherichia coli* BL21 were purified on glutathione-Sepharose 4B beads (Pharmacia, Piscataway, NJ, USA) according to the manufacturer’s instructions. Using the TNT T7 rabbit reticulocyte Quick Coupled Transcription/Translation system (Promega, San Luis Obispo, CA, USA), the ^35^S-labelled *in vitro* translation products of pcDNA3.1-human APLP1 and matriptase were prepared in the presence of [^35^S] EasyTag EXPRESS labelling mix (73% Met/22% Cys; 41,000 Ci/mmol, Perkin Elmer). The *in vitro* translated products were incubated with 10 μg of purified GST or GST-fusion protein in 20 mM Tris–HCl buffer (pH 7.4) containing 150 mM NaCl, 1% triton x-100, protease inhibitors for 2 h at 4 °C. Beads were washed four times with the same buffer. Bound proteins were eluted with Laemmli buffer, resolved by SDS–PAGE and visualized by autoradiography.

### Mass spectrometry analysis

GST fusion proteins were expressed in *Escherichia coli* BL21 and purified on glutathione-Sepharose 4B beads (Pharmacia, Piscataway, NJ, USA) according to the manufacturer’s instructions. For 2 hours at 37 °C, bound proteins were incubated in 100 μL of 100 mM Tris-HCl (pH 8.5) containing or not 100 nM of recombinant soluble WT matriptase. The supernatant was then collected, lyophilised and suspended in 25 μL of 10 mM HEPES/KOH (pH7.5). As previously described^38^, mass spectrometry analysis was performed using an Orbitrap QExactive mass spectrometer (Thermo Fisher Scientific) and data were processed, searched, analyzed, and quantified using the MaxQuant software package version 1.5.2.8 employing the Human Uniprot database (16/07/2013, 88,354 entries).

### Treatment of cells with Recombinant Matriptase

Cleavage assays *in cellulo* using purified recombinant matriptase were performed as described previously^38^. Briefly, culture medium of HEK293T was removed and replaced with 2 mL of serum-free HCELL-100 medium (Wisent, St-Bruno, QC, Canada) containing 5 nM of recombinant soluble human WT matriptase or mutant S805A. The conditioned medium was collected after 36 h incubation and concentrated with Amicon Ultra Centrifugal filters 3,000 NMWL (Merck Millipore Ltd., T., C., Co Cork IRL). Both cell lysates and concentrated conditioned medium were then analyzed by SDS-PAGE and immunoblotting.

### Statistical analysis

Experiments were performed at least in triplicate and a one sample t-test with a hypothetical value set to 1 for GST alone was used for GST-Pull Down quantification analysis. A two-way ANOVA with a Sidak’s multiple comparisons test was used in BRET assay.

## Supplementary information

Supplementary information.
