# Supplementary material for: Matriptase processing of APLP1 ectodomain alters its homodimerization
Source: Sci Rep. 2020 Jun 22;10:10091. doi: 10.1038/s41598-020-67005-6 (PMC7308337; doi:10.1038/s41598-020-67005-6)
Supplement: Supplementary file 1 — Supplementary information. [file 41598_2020_67005_MOESM1_ESM.docx]

**Matriptase processing of APLP1 ectodomains alter its homodimerization**

**Erwan Lanchec, Antoine Désilets, François Béliveau, Cloé Fontaine-Carbonneau, Andréanne Laniel Richard Leduc* and Christine Lavoie***

**Supplementary Information**

Table of contents:

FIGURE S1. Matriptase interacts with GFP-APLP1 but not with GFP or VAMP8-GFP p2

FIGURE S2. Matriptase interacts with APLP1-Flag. P3

FIGURE S3. In vitro interaction of matriptase with the ectodomain of APLP1 p4

FIGURE S4. Matriptase cleaves APLP1 in cellulo p5

FIGURE S5. Matriptase cleavage of APLP1 is inhibited by HAI-1 p6

FIGURE S6. Matriptase-2 does not cleave APLP1 p7

FIGURE S7. Matriptase cleaves APLP1 at arginine 124 p8

FIGURE S8. Sequence alignment of APP and APLP1 p9

**Supplementary Figure S1**

**Supplementary Figure S1.** **Matriptase interacts with GFP-APLP1 but not with GFP or GFP-VAMP8.** Lysate of HEK293 cells transfected with matriptase and GFP-tagged APLP1, luminal GFP or VAMP8-GFP were immunoprecipitated (IP) with GFP-Trap beads and then immunoblotted (IB) with anti-matriptase, anti-actin or anti-GFP antibodies to detect matriptase, GFP, VAMP8, actin and APLP1, respectively. Full length matriptase is detected as a doublet at 95 KDa (n=3).

**Supplementary Figure S2**

**Supplementary Figure S2. Matriptase interacts with APLP1-Flag but not with Flag alone.** Lysate of HEK293 cells transfected with matriptase and APLP1-Flag or Flag were immunoprecipitated (IP) with Flag antibodies and then immunoblotted (IB) with anti-matriptase or anti-Flag antibodies to detect matriptase and APLP1-Flag, respectively. Full length matriptase is detected as a doublet at 95 KDa (n=3).

**Supplementary Figure S3**

**Supplementary Figure S3. *In vitro* interaction of matriptase with the ectodomain of APLP1**. GST protein or GST-tagged APLP1 ectodomain (N-term) or cytosolic domain (C-term) (10 μg) were immobilized on glutathione beads and incubated with *in vitro* translated ^35^S-labeled matriptase. Bound proteins were separated by SDS-PAGE and detected by autoradiography (upper panel). GST proteins were detected with Coomassie blue staining (lower panel). Input = 2.5% of the total *in vitro* translated product (n=3).

**Supplementary Figure S4**

**Supplementary Figure S4. Matriptase cleaves APLP1 *in cellulo*.** HEK293 cells were transfected with WT matriptase, a catalytically inactive matriptase mutant (S805A) or empty vector (mock) together with GFP-tagged APLP1. Lysates and conditioned media were immunoblotted with anti-matriptase or anti-GFP antibody to detect matriptase, APLP1 and APLP1 fragments (n=3 for each APLPs). A GFP-tagged APLP1 fragment (cleaved) of 33 kDa is detected in cell lysate and medium (arrow). Full length and catalytic domain of matriptase are detected at 95 KDa and 30 KDa, respectively (n=3).

**Supplementary Figure S5**

**Supplementary Figure S5.** Matriptase cleavage of APLP1 is inhibited by HAI-1**.** Lysate of HEK293 cells transfected with GFP-tagged APLP1 and matriptase together with HAI-1 or empty vector (mock) were immunoblotted with anti-matriptase, anti-HAI-1 and anti-GFP antibody to detect matriptase, HAI-1, APLP1 full-length and APLP1 fragments (n=3).

**Supplementary Figure S6**

**Supplementary Figure S6.** Matriptase-2 does not cleave APLP1. Lysate of HEK293 cells transfected with GFP-tagged APLP1 together with wild-type (WT) matriptase (Mat-WT), a catalytically inactive matriptase mutant (Mat-S805A), V5-tagged matriptase 2 (Mat2-WT) or empty vector (mock) were immunoblotted with anti-matriptase, anti-V5 or anti-GFP antibodies to detect matriptase, matriptase-2, and APLP1 full-length/fragment, respectively (n=3).

**Supplementary Figure S7**

**Supplementary Figure S7**. Matriptase cleaves APLP1 at arginine 124**.**Tandem mass spectrometry spectra from peptide ion trap CID fragmentation for the peptide SGSCAHPHHQVVPF identified after *in vitro* digestion and LC-MS/MS analysis of GST-APLP1 ectodomain fragments generated by matriptase cleavage. Shown is a representative annotated MS/MS fragmentation spectrum with the identified matched amino terminus-containing ions (b ions) in blue and the carboxyl terminus-containing ions (y ions) in red. Peptide intensities were summarized per amino acid residue and plotted in relation to each other. The detected peptide sequences indicate that Arg124 is the main cleavage site.

**Supplementary Figure S8**

**Supplementary Figure S8**. Sequence alignment of the cleavage sequence in the E1 domain of APP and APLP1.
